# An Experimental Study on Bubble Growth in Laponite RD as Thixotropic Yield Material

**DOI:** 10.3390/ma13132887

**Published:** 2020-06-27

**Authors:** Yiping Zhang, Mengxian Hu, Yongchao Zhou

**Affiliations:** College of Civil Engineering and Architecture, Zhejiang University, Hangzhou 310058, Zhejiang, China; zhangyiping@zju.edu.cn (Y.Z.); humengxian@zju.edu.cn (M.H.)

**Keywords:** Laponite RD, bubble growth, bubble morphology, yield stress, dimensionless number

## Abstract

The growth and release of the leading major bubble at the tip of a needle in the thixotropic yield material Laponite RD was different from subsequent minor bubbles. The gas injection experiments combined with high-speed camera were conducted. The results showed that the shape of the major bubbles transformed from an inverted carrot shape to an inverted teardrop shape, while the shape of the minor bubbles tended to be elliptical. In addition, the pressure of bubble emergence consisted of hydrostatic pressure, capillary pressure, and cracking pressure. The major and minor bubbles differed only in the cracking pressure. The pressure when the minor bubble detached could be estimated from the lateral hydrostatic pressure. It can be deduced from dimensionless numbers that buoyancy and viscous forces were, respectively, the main driving force and resistance of bubble growth. The yield stress of Laponite RD and inertial force at the initial moment resulted in distinctive behavior of the major bubble. In addition to the viscosity resistance, surface tension, and hydrostatic pressure had a non-negligible influence on minor bubbles and still accounted for 10–20% of the total resistance in the later stage but less than 5% in major bubble growth.

## 1. Introduction

Bubbling behavior is a fundamental problem of two-phase gas–liquid flow and is widely encountered in various engineering applications, especially in the fields of energy, chemicals, and environment such as methanol fuel cells [1,2], sewage treatment [3,4], frictional drag reduction [5,6], and gas–liquid contactors [7,8]. The laws governing the size, shape, and motion of bubbles are important factors affecting the flow characteristics of a two-phase gas–liquid system and also an important basis for the design and operation of gas–liquid mass transfer equipment and chemical reactors. Bubble dynamics are the key link when attempting to solve the above problems, as they can be applied to predicting the total gas emissions and the shape of bubbles during the gas release process.

Extensive research has been conducted in the form of experiments and numerical analysis of bubble morphology. Kulkarni and Joshi [9] reviewed the bubble formation and speed of ascent in a gas–liquid system and comprehensively described the bubble growth and motion in Newtonian fluids. It is worth noting that bubbles on the scale of a few micrometers to a few hundred micrometers in Newtonian fluids are relatively easier to study, because they are almost spherical shape and not easily deformed. For large bubbles with a radius of more than 1000 microns, the shape is usually distorted and no longer spherical [10]. Furthermore, most of the fluids encountered in industrial processes are non-Newtonian fluids such as polymer solutions and melts, fermentation broths, sludges, and cosmetics. Compared with Newtonian fluids, bubble motion in non-Newtonian fluids becomes more complicated and difficult to predict due to the complex rheology. Some scholars have studied the bubbling behavior of a single gas escaping from the orifice of non-Newtonian fluids and found that the formation of orifice bubbles was affected by the physical properties of the fluid including: viscosity, density, surface tension, etc. [11,12]. Snabre and Magnifotcham [13] considered the continuous emission of gas bubbles from a single ejection orifice immersed in water–glycerol systems and analyzed the effects of fluid viscosity, gas flow rate, orifice diameter, and liquid depth on the bubble stream dynamic to derive an expression for the net viscous force acting on the surrounding fluid. Vafaei and Wen [14,15] injected air into deionized water and nanofluids to observe the effect of nanoparticles on the three-phase contact line and bubble morphology, and used the Young–Laplace equation to predict the evolution of bubbles. It was found that within a few milliseconds before the bubble detaches, the Young–Laplace equation could no longer accurately describe the real-time boundary of the bubble. Terasaka et al. [16,17,18] investigated the effects of gas chamber volume, nozzle diameter, gas flow rate, and fluid rheology on bubble volume in glycerin, polyacrylamide aqueous solution (PAA), and Xanthan gum solution, and proposed a growth model of aspherical bubbles based on the Rayleigh–Plesset equation. Bubble volume and shape, and pressure fluctuations in the gas chamber were predicted relatively well by the present model. Both Sikorski et al. [19] and Lopez et al. [20] studied the effects of rheology and bubble size on bubble velocity and shape in the viscoelastic material Carbopol gel, and found that the bubble had an “inverted teardrop” shape; they believed that the elasticity and yield stress effects played an important role in the shape of the bubbles. Kee et al. [21], examined air, nitrogen, and carbon dioxide bubbles in solutions of polyacrylamide in 50/50 glycerine/water mixtures under a wide range of conditions, and suggested that the fluid viscoelasticity influences the coalescence via the shapes and wake characteristics of the bubbles. The study of Samson et al., also showed that under quasi-static conditions, the bubble geometry was related to the internal pressure of the bubble, the rheological behavior, and the yield stress of the Carbopol gel. A modification of the Laplace law depending on the yield stress of the fluid and bubble sphericity was proposed and validated [22].

In previous research, materials such as Carbopol gel and Xanthan gum were mainly used as the non-Newtonian fluids. They are typical yield stress fluids with no thixotropy [23]. Thixotropic yield stress fluids usually have a relatively stable internal structure, and can be widely used as a thixotropic agent, thickener, dispersant, suspending agent, and stabilizer in fine chemicals, light industry, daily chemical industry, and medicine. As a representative thixotropic yield stress fluid, Laponite RD is a synthetic layered silicate and is also considered to be one of the best materials for the study of clay dispersion systems, which can be used to make transparent clay that simulates “very soft marine clay” [24]. Some scholars proposed using Laponite RD as an alternative to sewer sediments at the end of the last century [25]. Several of scholars subsequently applied Laponite RD to simulate engineering soft clay or marine sediments in the fields of geotechnical and marine engineering, combined with digital imaging, particle tracking, and other technologies to observe the deformation of the material due to destruction [26,27], gas–liquid hydrate release route [28], and crack propagation trajectory [29,30]. Zhang et al. [31] conducted a systematic study on the rheological properties of Laponite RD, tested its viscoelasticity, and established a viscosity model. The lack of experimental research into the growth of bubbles in Laponite RD also hinders its further application.

For the thixotropic yield stress fluid, the growth process and morphological characteristics of the first bubble are significantly different from the subsequent bubbles. The first bubble also affects the growth and rise of the subsequent bubbles. The growth of the subsequent bubbles has a relatively stable periodicity. At present, most research works in the field of bubble morphology is centered on the shape, rising trajectory, and final velocity of subsequent periodic bubbles. Byakova et al. [32] proposed a four-stage theory of bubble growth stage of bubbles (nucleation, subcritical growth, critical growth, and necking), which is also aimed at the stable growth of bubbles. Zhu et al. [33] divided the bubble formation process into three stages: the initial growth, the speed-up growth, and the speed-down growth. However, there is still a lack of detailed discussion on the growth process of the first bubble. Therefore, the current understanding of the generating conditions necessary for generation and growth stages of the first bubble remains unclear.

In this study, gas injection tests into the Laponite RD were conducted. High-speed camera and digital image processing technologies were used to compare the growth and morphological characteristics of the first bubble (hereinafter referred to as the major bubble), as well as subsequent periodic bubbles (referred to as the minor bubbles). An outline of the paper is as follows. Section 2 presents the experimental materials and methods. The results obtained are presented and discussed in Section 3. A detailed analysis of the variations of parameters such as morphology and pressure, dimensionless number and force of major and minor bubbles during the entire gas injection process are discussed in Section 3.1, Section 3.2 and Section 3.3, respectively. The paper ends with Section 4 with a few final remarks.

## 2. Materials and Methods

### 2.1. Preparation of Laponite RD

Laponite RD powder was a synthetic silicate with the same refractive index as water. It could be rapidly dispersed in water into a disk-like shape with a diameter of about 25 nm and a thickness of about 1 nm, forming a colorless and transparent suspension [34]. Laponite RD powder was manufactured by Southern Clay Products Inc. (TX, USA). The powder was slowly added to the vigorously stirred water to ensure uniform dispersion and avoid flocculation. The deionized water was used to prevent the ion coagulation of the powder and improve transparency. The blender was then placed in a vacuum tank for a fully stirring under vacuum condition to remove the entrapped air bubbles. Laponite RD with a concentration of 3% (powder weight/total weight) was set for 2 days. After the sample achieved a transparent and stable state, the gas injection tests were conducted.

The viscosity of 3% Laponite RD conforms to the Herschel–Bulkley model, and the values of the parameters related to the material used in this article are shown in Table 1:

(1) $$\begin{array}{c} \eta_{c}=\eta \left(\dot{\gamma }\right)=\tau_{y}{\dot{\gamma }}^{-1}+K{\dot{\gamma }}^{n-1} \end{array}$$

### 2.2. Experimental Apparatus and Methods

**Figure 1:**
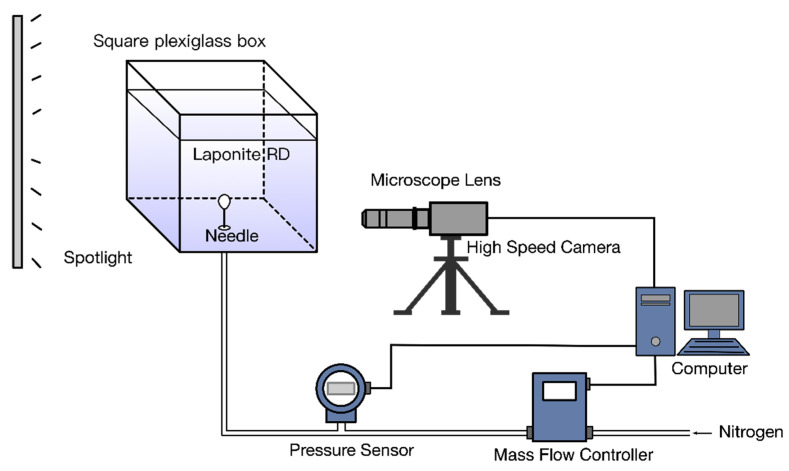
Schematic of experimental apparatus.

Figure 1 is a schematic diagram of the gas injection test system. Laponite RD sample was placed in a square plexiglass box. Its cross-sectional size was 100 mm × 100 mm, and the sample height was 120 mm. The upper part was connected with the atmosphere. There was a small hole set on the center of the bottom of the container for installation of the needle. The outer diameter and inner diameter ($D_{0}$) of the needle was 0.82 mm and 0.51 mm, respectively. The 10 mm part of needle extended was insert into the sample. Pure nitrogen gas was injected into Laponite RD through the pipe and needle, and the density of nitrogen gas was $\rho_{g}=1.17\;\mathrm{kg}/m^{3}$. The gas mass flow controller (Sevenstar^®^ CS200A, Beijing, China) controlled the stable flow rate $Q=1.67\times 10^{-8}\;m^{3}/s$ by constant power sensor and temperature compensation technology. The total pipe volume of the gas injection system was 15 mL verified by the ideal gas state equation and water infiltration method. The gas injection pressure was collected with a pressure transmitter (MEACON MIK-P300, Hangzhou, China) at a frequency of 1 kHz. A high-speed camera (FASTCAM SA5, San Diego, USA) at 1000 fps was used to capture and record the bubble growth process in real time. And resolution equals to 1024 × 1024 pixels. An ISO light sensitivity of 4000 (color) was measured to the published ISO 12232 standard. The lens used in experiments was a Nikon NIKKOR (Kyoto, Japan) 18–140 mm f/3.5–5.6G. A microscope lens (LWD-1000) was used to obtain the detailed images of the needle tip.

**Figure 2:**
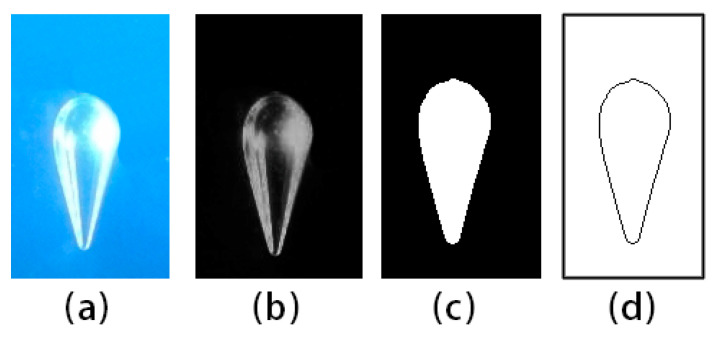
Steps of image processing: (**a**) initial image; (**b**) background division and binarization; (**c**) hole filling; (**d**) edge extraction.

The obtained bubble images were used to extract the characteristic parameters of the bubble shape by MATLAB_R2016a. Figure 2 shows a typical image and its postprocessed frames. Taking into account the camera pixel resolution and light scattering, the image process error was less than 5% in bubble edge extraction for bubble radius greater than 1.5 mm.

## 3. Results and Discussion

### 3.1. Morphology and Pressure during Bubble Growth

**Figure 3:**
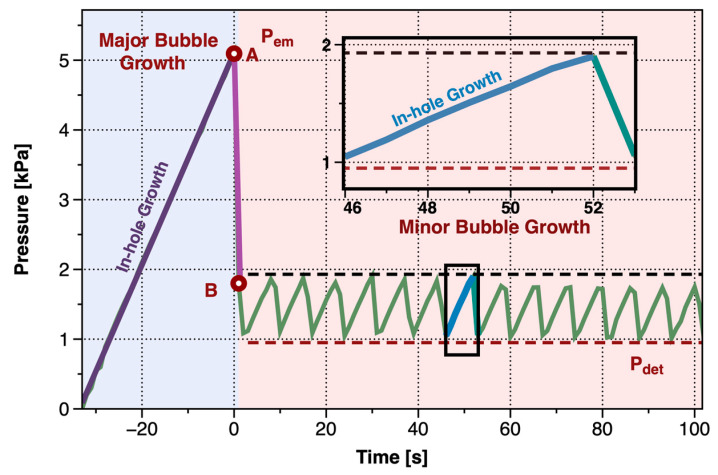
Pressure curve of the gas injection test.

The gas pressure change during the gas injection tests was shown in Figure 3, which was in a sawtooth shape that rose first and then fell. When the air pressure accumulated large enough to force the gas injected into the Laponite RD, it would generate bubbles in the soil that were visible to the naked eye. With the change of air pressure, the first bubble (called the major bubble) and the bubble generated in the subsequent cycle (called the minor bubble) had completely different morphological characteristics. The curve of air pressure could be divided into two parts, accordingly, the major bubble and the minor bubble. The major bubble contained in-hole growth, rapid vertical growth, and slow growth, while the minor bubble contained in-hole growth and slow growth.

#### 3.1.1. The In-Hole Growth

**Figure 4:**
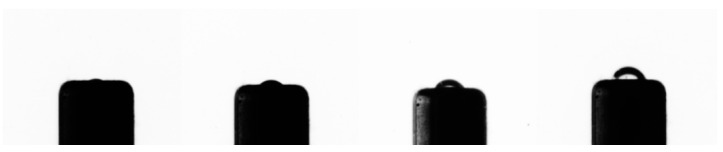
The process of the bubble emerging from the tip of the needle (photographed through a microscope lens).

Before the experiment began, the Laponite RD in the sample box was pressed into the needle tube due to the hydrostatic pressure, and the gas–liquid interface was located inside the needle [33]. As the gas was injected at a constant flow rate, the pressure in the gas injection tube and the needle increased linearly. The gas–liquid interface rose along the inner wall of the needle and gradually emerged from the top of the needle (Figure 4). This process was called the in-hole growth period. Byakova et al. [32] defined the stage where the bubble emerged from the needle until the diameter of the bubble was equal to the inner diameter of the needle as a nucleation phenomenon. The gas–liquid interface in this period developed slowly because of the influence of the hydrostatic pressure, capillary pressure, and the physical and mechanical properties of the overlying material. Therefore, it can be concluded that no visible bubbles were generated at this stage.

In the in-hole growth, the hydrostatic pressure $P_{h}=\rho_{l}gH$ and capillary pressure $P_{ca}=\frac{4\sigma }{D_{0}}$ were equilibrated with the air pressure, where H was the height from needle tip to liquid surface. The two items totaled 1.68 kPa, while the hydrostatic pressure was 1.1 kPa and the capillary pressure was 0.57 kPa. However, as can be seen from Figure 3, the maximum growth pressure of the major bubble reached $P_{em}=5.28\;\mathrm{kPa}$, which was much larger than the sum of hydrostatic pressure and capillary pressure. It indicated that there were other external pressure preventing the gas from entering the sample and promoting the accumulation of air pressure.

The particles inside Laponite RD would form a “house of cards” network structure to resist cracking [35]. In addition, it can be seen from the growth process of the major bubble that the wedge-shaped head was similar to cracks formed in solid material. Assuming that the pressure required for cracking was $P_{cr}$ and according to air pressure balance:

(2) $$\begin{array}{c} P_{em}=\rho_{l}gH+\frac{4\sigma }{D_{0}}+P_{cr} \end{array}$$

From this, the cracking pressure when the major bubble emerged was 3.5 kPa. In materials science, fracture toughness was the critical stress intensity factor of a crack or notch, where propagation of the crack or notch suddenly became rapid and unlimited. The critical value of stress intensity factor measured under plane strain conditions was known as the plane strain fracture toughness, denoted $K_{cr}$ [36]. In the light of the calculation method [37], the critical stress intensity factor ($K_{cr}$) of Laponite RD calculated from the crack pressure was $K_{cr}=1.04P_{cr}\sqrt{\frac{\pi D_{0}}{2\times 2.464}}=67.5\;\mathrm{Pa}\cdot m^{1/2}$. The value of $K_{cr}$ was in the same range as that reported in the literature for similar viscous material [38,39].

During the minor bubble growth, the peak value of air pressure was about 1.93 kPa on average. According to Equation (2), $P_{cr}=0.25\;\mathrm{kPa}$ and the corresponding fracture toughness was $K_{cr}=4.7\;\mathrm{Pa}\cdot m^{1/2}$, and the fracture toughness decreased to 7% of the original state. The reason was that the previous bubbles had caused shear disturbance to the sample, and the subsequent bubbles only needed to overcome the residual strength recovered by the channel healing after the rising of previous bubbles. Compared with the fracture toughness before the disturbance, the remaining fracture toughness was almost negligible.

#### 3.1.2. The Major Bubble Growth

**Figure 5:**
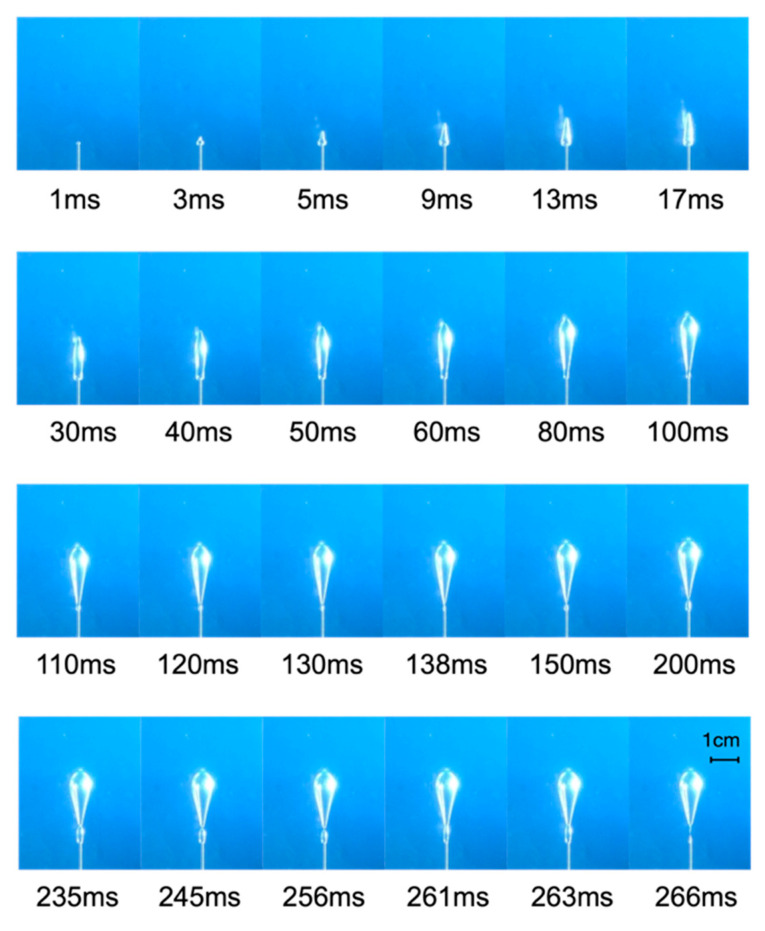
The major bubble growth process.

The major bubble took a short time from appearance to escapement (~266 ms), and the pressure dropped sharply from the maximum value $P_{em}$, corresponding to the segment A–B in Figure 3. Figure 5 showed the morphological changes during the growth of the major bubble observed by the high-speed camera.

**Figure 6:**
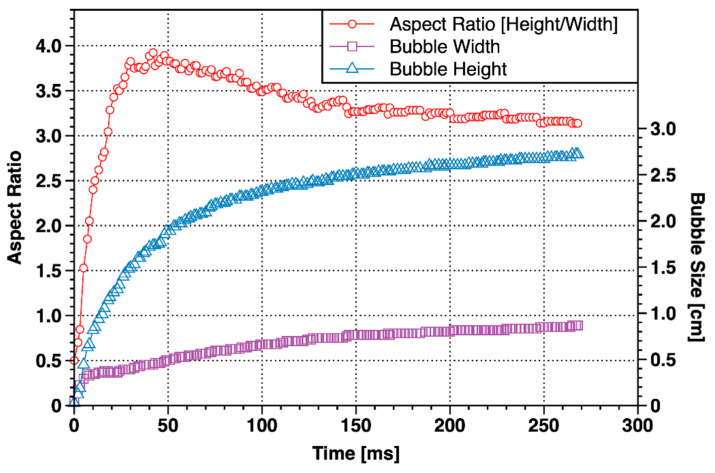
Changes in shape of the major bubble with time.

As can be seen from Figure 5, from the appearance of bubbles (0 ms) to about 40 ms, the bubbles were mainly in the shape of inverted “carrots” with small head and large bottom. The bubble was quickly “punctured” into the Laponite RD with a wedge-shaped head. At this stage, the height of the bubble increased sharply, much faster than the increase of width, and the aspect ratio of the bubble also increased quickly, reaching a peak at about 40 ms, as shown in Figure 6.

From 40 ms to 138 ms, the wedge-shaped head of the bubble gradually changed to a hemispherical shape, and the shape of the bubble gradually transformed to an inverted teardrop shape with a large head and a small bottom. The bubble width increased obviously while the aspect ratio gradually decreases. At the end of this stage, the tail of the bubble necked, and the bubble tended to move away from the needle tip. After 138 ms, a tailing bubble was formed between the major bubble and the needle. The shape of the major bubble remained unchanged, and the aspect ratio tended to be stable. There was still a small channel connecting the major bubble and the tailing bubble. The major bubble and the tailing bubble completely separated when the tailing bubble expanded to 245 ms. Then (to 261 ms) the tailing bubble “caught up” and merged with the major bubble in no time and became a single bubble at 266 ms. When the single bubble thoroughly escaped from the needle tip, the next bubble growth period would start. In other parallel tests, we also observed the situation with no tailing bubbles. It indicated that the tailing bubbles generated at this stage were not an inevitable phenomenon but were caused by the unobstructed channel of gas into the major bubble.

In summary, the growth of the major bubble could be divided into two stages. The first stage was the rapid vertical growth stage (0~40 ms), where the bubble took the form of an inverted carrot. The second stage was the slow expansion stage (after 40 ms); the final shape of the bubble was an inverted teardrop.

#### 3.1.3. The Minor Bubble Growth

**Figure 7:**
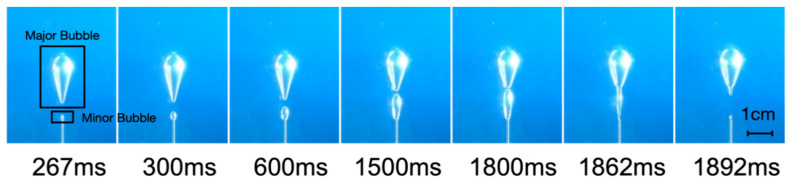
The minor bubble growth process.

**Figure 8:**
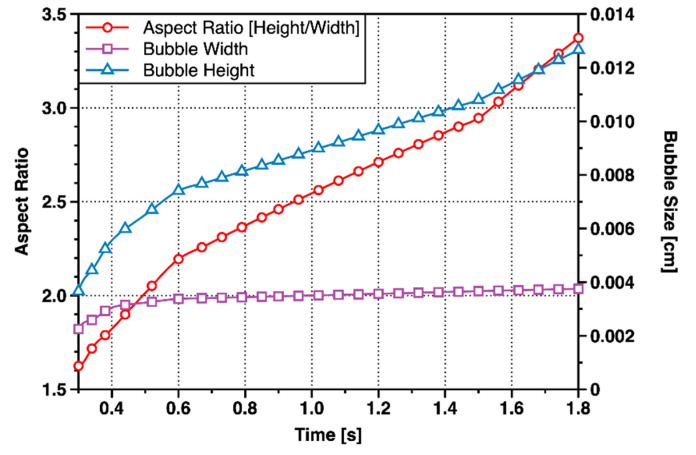
Changes in shape of the minor bubble with time.

The pressure of the system dropped from the maximum value of 5.28 kPa to about 1.9 kPa when the major bubble matured. Because the sample had been disturbed by the major bubble, the strength of the sample declined significantly due to the thixotropy of Laponite RD. But the air pressure was still enough to “squeeze” the gas into the sample at this time. Therefore, a new bubble (referred to as the minor bubble) appeared immediately after the major bubble separated from the needle tip. Compared with the major bubble, the generation process of the minor bubble was significantly different (as shown in Figure 7):

(1) The minor bubble growth time reached 1.62 s, which was about six times the growth time of the major bubble. There was no stage of rapid vertical growth stage, and the growth rate of bubble height and width was much smaller than the major bubble (Figure 8), but the final aspect ratio was approximately 3.4 in both major bubble and minor bubble.

(2) The size of the minor bubbles was evidently smaller than that of the major bubbles. The volume of the bubble after maturation was 0.094 mL, while the major bubble was 0.77 mL, which was about eight times larger than the minor bubble.

(3) The shape of the minor bubble tended to be in an ellipsoidal shape, rather than the inverted teardrop shape of the major bubble.

(4) Minor bubbles were generated and escaped one by one through the needle tip in a stable cycle with a period of 7.62 s, where the pressure accumulation time was about 6 s and the bubble growth time was 1.62 s.

After the major bubble detached from the tip of the needle, without the continuous injection of gas, it would stay in the yielding fluid and nearly “wait” for the minor bubble to merge. The rise rate of the major bubble was much smaller than that of the minor bubble. As the minor bubble gradually expanded, it would come into contact with the leading major bubble directly and merged into the major bubble (as shown from Figure 7). It was possible that the minor bubble slowly rose to the upper boundary and caught up with the major bubble after detachment. We observed both phenomena in our experiments.

#### 3.1.4. Calculation of Pressure at the Detachment Point of the Minor Bubble

When the pressure of the system dropped to approximately 0.95 kPa, the pressure was no longer able to squeeze the gas into the bubble. The channel formed by the rise of the bubbles healed, and the minor bubbles detached (or annihilated). During the periodic stage, the pressure valleys were all basically the same, $P_{det}=0.95\;\mathrm{kPa}$, corresponding to the red, dashed line in Figure 3.

**Figure 9:**
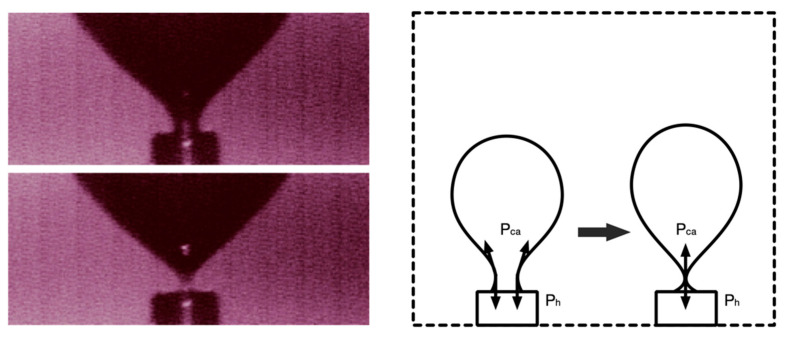
The process of bubble necking (photographed through the microscope lens) and schematic diagram.

At the end of the entire growth process, bubble detachment occurred when the air pressure was at its lowest, and the tail of the bubble would neck at the same time. At the necking, the surface tension was distributed vertically, and the horizontal component was zero (Figure 9). Therefore, when the air pressure dropped to balance with the horizontal lateral pressure of the Laponite RD (i.e., the net horizontal pressure was 0), it was the condition for the bubbles to detach. The air pressure when the bubble detached should be:

(3) $$\begin{array}{c} P_{det}=K_{0}\rho_{l}gH \end{array}$$

$K_{0}$ was lateral pressure coefficient of Laponite RD and it can be calculated by Poisson’s ratio, $K_{0}=\frac{v}{1-v}$. The pressure was calculated as 0.9 kPa from Equation (3). Considering that the strength of the soil decreased after the disturbance, the lateral soil pressure coefficient will increase, so the actual pressure was slightly larger (the actual value was 0.95 kPa). Hence, it is reasonable (slightly smaller) to estimate the gas pressure when the bubble detached according to Equation (3).

After detachment of the last bubble, the pressure accumulated again to about 1.9 kPa, and new bubble would reappear. The growth process and shape of newly emerged bubble were basically the same as previous minor bubbles.

### 3.2. Dimensionless Numbers

**Figure 10:**
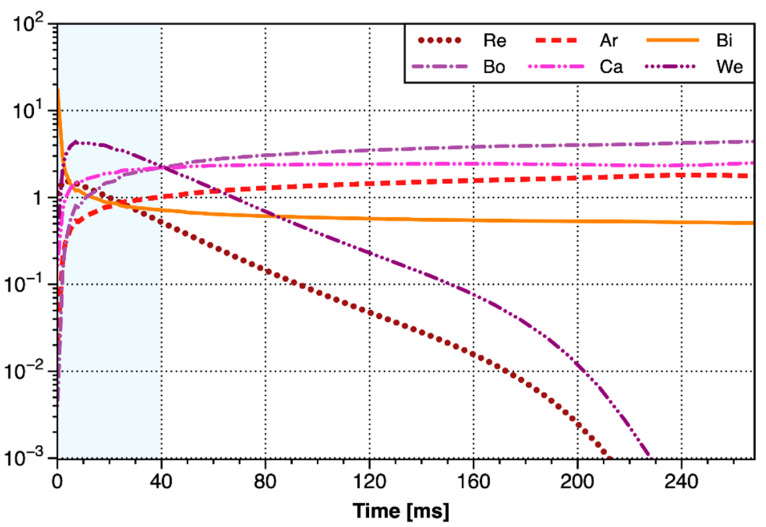
The curves of the dimensionless number of the major bubble with time.

During the growth process, the bubbles were subjected to a variety of forces to form different shapes. The relative magnitude of the force mainly depended on the size of the bubbles and the characteristics of the fluid. As shown in Table 2, these dimensionless numbers were used to describe the relative magnitudes of different forces, and their changes with time were shown in Figure 10.

Bingham number, capillary number, Weber number characterized the competition between buoyancy, viscous, and inertial forces with surface tension, respectively. It can be seen from Figure 9, that Bo and Ca increased rapidly within the initial 2~3 ms and then increased slowly, indicating that the surface tension only had an influence within the initial few milliseconds. As the bubbles grew, buoyancy and viscous force soon dominated. In the second stage of the growth of the major bubble (after 40 ms), Bo gradually exceeded Ca, indicating that the buoyancy began to be greater than the viscous resistance, and the bubble began to rise. The tail of bubble gradually necked down due to the rise, and finally the bubble detached from the needle tip. Weber number as almost the largest number in the first stage of the major bubble, even exceeding the effect of surface tension especially in the first few milliseconds. In the second growth stage of the major bubble, the acceleration of bubble growth decreased, and We dropped sharply, indicating that the inertial force was no longer significant at this time. Reynolds number and Archimedes number characterized the competitive relationship between inertial force, buoyancy force with viscous force, respectively. The variations of Re was similar to We, and it acted mainly on the first stage of bubble growth, emphasizing that the inertial force had a non-negligible effect only in the first stage. Except for the initial few milliseconds, Re was always less than We, indicating that the viscous force was greater than the surface tension. Bingham number represented the comparison between the yield stress of the fluid and the inertial force. As can be seen from Figure 9, Bi was very large at the initial stage, indicating that the main resistance to be overcome was the yield stress when the bubble emerged. It was consistent with the fact that the cracking pressure accounted for nearly 70% of the maximum pressure. After a sharp drop in the initial milliseconds, Bi began to decrease slowly to slightly less than 1, indicating that the effect of yield stress and the effect of inertial force thereafter were basically equivalent.

Based on the analysis above, in the first stage of bubble growth, the resistance forces in the first few milliseconds were mainly the yield stress, followed by the inertial force and surface tension. Soon the effect of surface tension became almost negligible, and then the mainly resistance forces became the viscosity force, followed by the inertial force. In the second stage of bubble growth, the viscous force was the dominating resistance force, and the remaining resistance forces were almost negligible.

**Figure 11:**
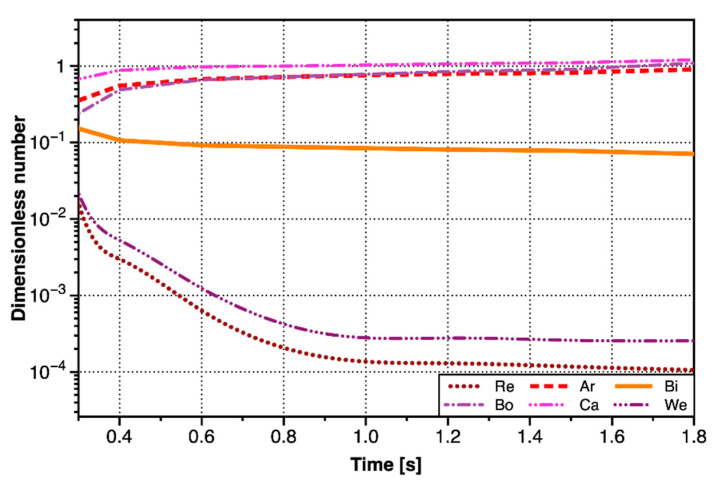
The curves of the dimensionless number of the minor bubble with time.

Since a major bubble had been generated and detached before, the growth environment of the minor bubble was the disturbed Laponite RD. It had been calculated above that the critical fracture toughness dropped to about 7% of the original value. Yield stress and shear modulus would also decrease due to the changes in the properties of Laponite RD. The values of $\tau_{y}$ proportionally took up 7% of the original values when calculating dimensionless numbers, because the indexes after disturbance could not be measured in real time. It was noted that the values of $\tau_{y}$ in the calculation of $\eta_{c}$ did not need to reduce, because the values of $\dot{\gamma }$ were still within the range of the model of $\eta_{c}$. The curves of the dimensionless number of the minor bubbles with time was shown in Figure 11. Its variation rules were significantly different from that of the major bubble, because there was no obvious staged change.

As can be seen from Figure 11, Ca>Bo≫We, indicating that the inertial force could be ignored. Bond number as slightly smaller than capillary number, indicating that the viscous force would play a more important role than buoyancy force. Archimedes number was closed to Bond number, while Archimedes number was greater than Bond number in the early stage, and opposite in the later stage. It indicated that surface tension was more important when the bubble volume was smaller, and the viscous force was the main influence factor in the later stage. Bingham number was also stable at about 0.1, indicating that the yield stress of Laponite RD had been greatly reduced, and the yield stress could be ignored. Therefore, in the minor bubble growth stage, the bubbles grew mainly to overcome the viscous force and surface tension. The minor bubble was small-sized, and the surface tension played an important role in controlling the bubble shape to be ellipsoid.

### 3.3. Analysis on Contacting Forces

**Figure 12:**
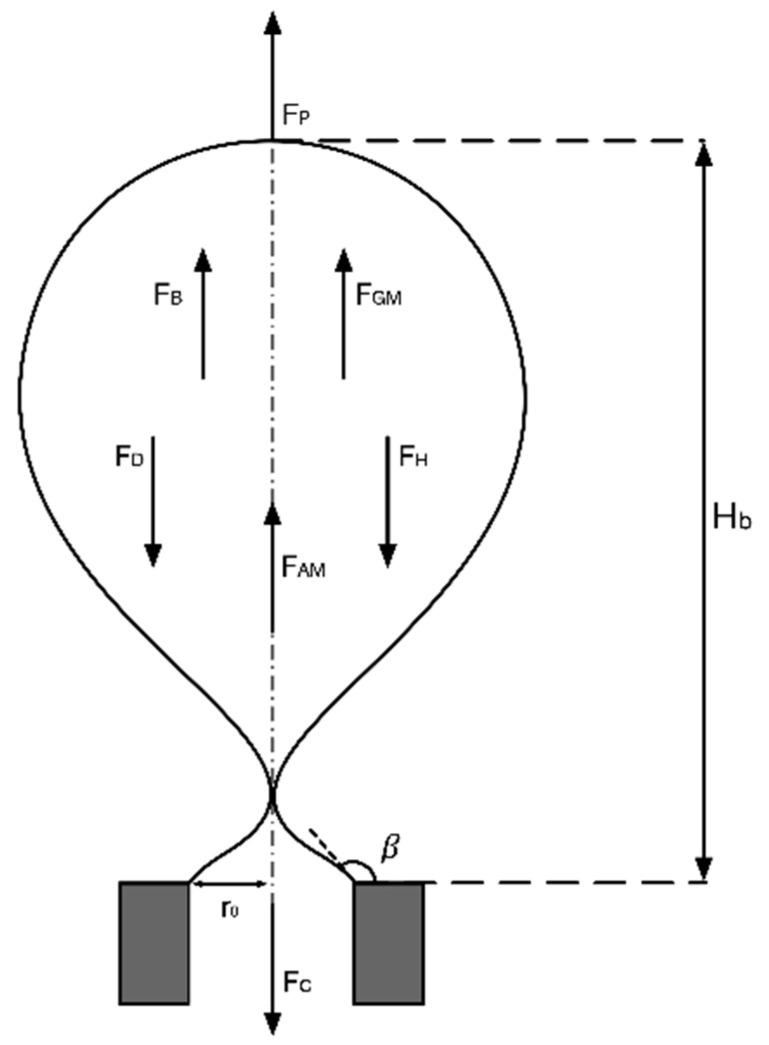
Schematic diagram of force balance.

The bubble formation in the orifice was mainly affected by buoyancy force, Young–Laplace pressure force, surface tension force, hydrostatic force, viscous drag force and gas momentum force [40,41], and the calculation equation of these forces were shown in Table 3. The joint action of forces determined the morphology of the bubble formation process and the bubble detachment volume. The bubble was regarded as a force isolator, and its effective force was shown in Figure 12:

According to Newton’s second law, the vertical motion equation of the bubble was established:

(4) $$\sum F=F_{B}+F_{P}+F_{GM}+F_{H}+F_{c}+F_{D}+F_{AM}=0$$

Except for the viscous drag force, other forces were calculated based on the size and shape of the bubbles and the performance parameters of the transparent clay, and then the viscous drag force can be calculated by Equation (4).

**Figure 13:**
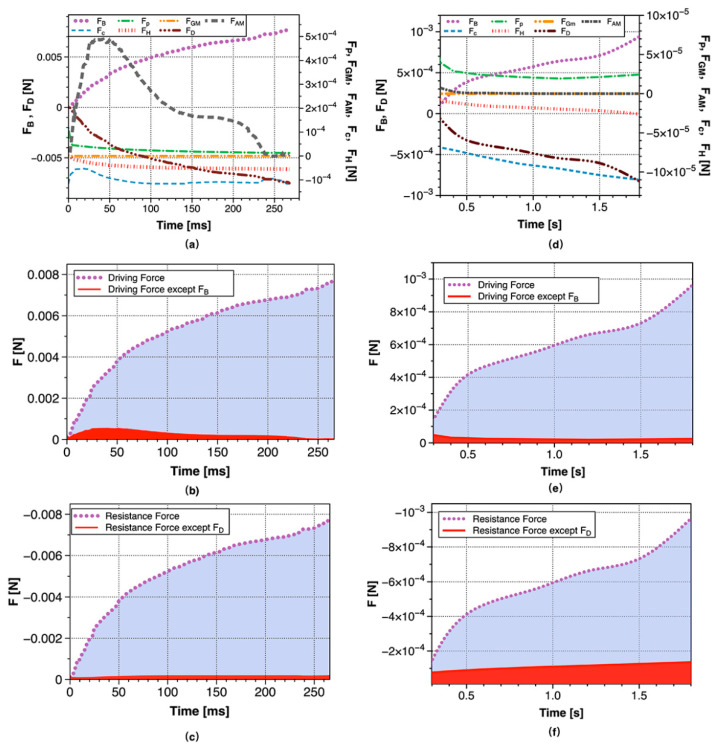
Curves of various forces with time in the major bubble growth (**a**–**c**) and minor bubble growth (**d**–**f**).

**Figure 14:**
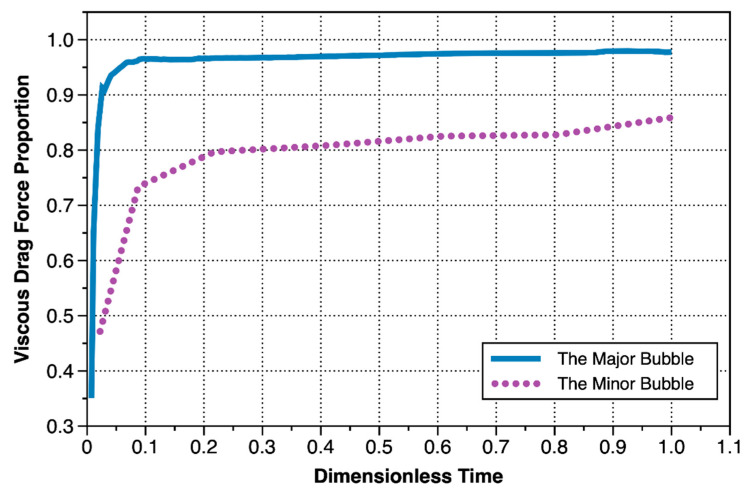
Proportion of viscous drag force at bubble growth stage.

The changes of forces applying on the major bubble with time were shown in Figure 13a. The forces that driving the bubbles upward included buoyancy force, Young–Laplace pressure force, gas momentum force and inertial force. As can be seen from Figure 13b, the proportion of Young–Laplace pressure force and gas momentum force were very small and could be negligible. The resistance force included viscous resistance, surface tension force, and hydrostatic force. It can be seen from Figure 13a that the inertial force gradually increased to the maximum in the first stage of the growth of the major bubble, playing a non-negligible role. After that the inertial force began to decrease, and the influence was less than the effect of Young–Laplace pressure force at the end of the second stage. Throughout the bubble growth stage, the surface tension force and hydrostatic force did not change significantly, and the value was also small. It can be seen from Figure 13c, that the resistance was mainly the viscous drag force, and other resistances (sum of surface tension force, and hydrostatic pressure force) occupied a lower proportion (Figure 14).

Figure 13d showed the variation of various forces during the minor bubble growth process. It can be seen from the figure that the gas momentum force and the inertial force could be completely ignored. The driving force for the rising of the bubble was still mainly buoyancy force, but the Young–Laplace pressure force also had a bit contribution (Figure 13e). Among the forces resisting bubble rising, the effect of surface tension force was non-negligible, and the hydrostatic force also had a certain effect. In addition to the viscous resistance, the sum of surface tension and hydrostatic pressure occupied a non-negligible proportion in the resistance force (Figure 13f), and there was still 10–20% resistance came from surface tension force and hydrostatic force in the late stage of bubble growth (Figure 14). Because the periods of the major and minor bubbles were different, in order to better compare the changes in the entire period, dimensionless time was used. The dimensionless time was the current time/total cycle time.

## 4. Conclusions

Based on a visual gas injection test (injection rate was 1 mL/min, and the inner diameter of the needle was 0.51 mm), this paper analyzes the growth process of bubbles in the thixotropic yield material Laponite RD (concentration of 3%). Through an analysis of bubble shape parameters, gas injection pressure, dimensionless flow parameters, and force, the following conclusions can be obtained:

1. The growth process of the major bubble can be divided into three stages: in-hole growth, rapid vertical growth, and slow expansion. In the rapid vertical growth stage, dimensionless numbers *We*, *Re*, and *Bi* dominated, indicating that inertial force and yield stress were the major forces to bubble growth at this stage, and caused the major bubble to take the form of an inverted carrot. In the stage of slow growth, dimensionless numbers such as *Bo*, *Ar*, and *Ca* began to dominate, indicating that buoyancy mainly competed with viscous resistance, and the bubble developed into and t an inverted teardrop type. There was no obvious rapid growth stage of minor bubbles, and the growth rate of bubbles was much smaller than that of the major bubble. Furthermore, *Bo*, *Ar*, and *Ca* always dominated, and the competition of force was always dominated by the competition between buoyancy and viscous resistance. The shape was oblong. After the Laponite RD was disturbed by the major bubble, the yield stress dropped significantly, so subsequent minor bubbles were significantly smaller than the major bubble. As the Laponite RD concentration decreased, the yield stress also decreased, so it could be reasonably inferred that a lower Laponite RD concentration would result in smaller bubbles. On the contrary, a larger volume of bubbles was generated;
2. The undisturbed Laponite RD exhibited “solid-like” characteristics and had a high yield stress, which was also why the growth pressure in the pores of the major bubble was much higher than that in the minor bubbles. The pressure during the growth phase of the bubble hole can be estimated using the relevant theory of fracture mechanics (Equation (2)). According to Equation (2), it can also be reasonably inferred that if the inner diameter of the gas injection needle were to increase, the peak pressure would decrease, the gas outlet flow rate would decrease, and the bubble expansion rate would decrease. If the inside diameter were to decrease, the opposite changes would occur. When the minor bubble grows, the Laponite RD showed “fluid” characteristics, the pressure accumulation in the minor bubble hole was mainly governed by hydrostatic pressure and capillary force, and the air pressure during the detachment was mainly governed by hydrostatic pressure, which can be estimated according to Equation (3);
3. After the bubble cracked the sample, the main resistance to overcome was viscous force, whereas other resistance accounted for less than 5% of the total. During the growth of the minor bubble, in addition to the viscosity resistance, the surface tension and hydrostatic pressure had a non-negligible effect on the upward growth of the bubble. In the later stage of the bubble growth, the sum of the two still accounted for 10–20% of the total resistance.

## Figures and Tables

**Table 1:** Properties of 3% Laponite RD [31].

$\tau_{y}\left(Pa\right)$	$K\left(Pa.s^{n}\right)$	n	$G^{\prime }\left(Pa\right)$	σ(N/m)	v	$\rho_{l}\left(kg/m^{3}\right)$
28.81	2.835	0.3744	275	0.073	0.447	1020

**Table 2:** List of dimensionless parameters.

Symbol	Name	Expression	Meaning
Re	Reynolds number	$\frac{\rho_{l}v_{c}R_{eff}}{\eta_{c}}$	Ratio of inertial force to viscous force
Ar	Archimedes number	$\frac{\rho_{l}gR_{eff}^{2}}{v_{c}\eta_{c}}$	Ratio of buoyancy force to viscous force
Bi	Bingham number	$\frac{\tau_{y}}{\rho_{l}gR_{eff}}$	Ratio of yield stress to inertial force
Bo	Bond number	$\frac{\rho_{l}gR_{eff}^{2}}{\sigma }$	Ratio of buoyancy force to surface tension
Ca	Capillary number	$\frac{\eta_{c}v_{c}}{\sigma }$	Ratio of viscous force to surface tension
We	Weber number	$\frac{\rho_{l}v_{c}^{2}D_{eff}}{\sigma }$	Ratio of inertial force to surface tension

$R_{eff}$, $D_{eff}$ were the equivalent radius and diameter, $R_{eff}={\left(\frac{4V_{b}}{3\pi }\right)}^{\frac{1}{3}}$, $D_{eff}=2R_{eff}$, $V_{b}$ represented volume of bubble. The rest of parameters were shown in Table 1.

**Table 3:** List of forces.

Symbol	Name	Expression
$F_{B}$	Buoyancy force	$\left(\rho_{l}-\rho_{g}\right)gV_{b}$
$F_{P}$	Young–Laplace pressure force	$\frac{2\sigma }{R_{0}}\pi r_{0}^{2}$
$F_{GM}$	Gas momentum force	$\frac{\rho_{g}Q^{2}}{2\pi r_{0}^{2}}$
$F_{AM}$	Inertial force	$-\left(\rho_{g}+\frac{11}{16}\rho_{l}\right)V_{B}a$
$F_{H}$	Hydrostatic force	$-\left(\rho_{l}-\rho_{g}\right)gH_{b}\pi r_{0}^{2}$
$F_{c}$	Surface tension force	$-2\pi \sigma r_{0}sin\beta$
$F_{D}$	Viscous drag force	$-\frac{1}{2}\rho_{l}C_{D}\pi R_{eff}^{2}v_{c}^{2}$

where $r_{0}$, $R_{0}$, $H_{b}$, $C_{D}$, β, a were the inner radius of needle, curvature radius of apex, bubble height, drag coefficient, contact angle, bubble average acceleration, respectively.

## Abbreviations

**Notation**	
*a*	bubble average acceleration (m/s^2^)
*Ar*	Archimedes number (-)
*Bi*	Bingham number (-)
*Bo*	Bond number (-)
*C_D_*	drag coefficient (-)
*Ca*	capillary number (-)
*D_0_*	inner diameter of needle (m)
*D_eff_*	equivalent diameter (m)
*F_AM_*	inertial force (N)
*F_B_*	buoyancy force (N)
*F_c_*	surface tension force (N)
*F_D_*	viscous drag force (N)
*F_GM_*	gas momentum force (N)
*F_H_*	hydrostatic pressure force (N)
*F_P_*	Young–Laplace force (N)
*g*	gravitational acceleration (m/s^2^)
*G*′	shear modulus (Pa)
*H*	height from needle tip to liquid surface (m)
*H_b_*	bubble height (m)
*K*	consistency index (Pa·s^n^)
*K_0_*	lateral pressure coefficient (-)
*K_cr_*	critical stress intensity factor (Pa·m^1/2^)
*n*	power law index (-)
*P_ca_*	capillary pressure (Pa)
*P_cr_*	crack pressure (Pa)
*P_det_*	pressure of bubble detachment (Pa)
*P_em_*	pressure of bubble emergence (Pa)
*P_h_*	hydrostatic pressure (Pa)
*Q*	gas flow rate (m^3^/s)
*r_0_*	inner radius of needle (m)
*R_0_*	curvature radius of apex (m)
*R_eff_*	equivalent radius (m)
*Re*	Reynolds number (-)
*V*	Possion’s ratio (-)
*v_c_*	centroid velocity (m/s)
*V_B_*	bubble volume (m^3^)
*We*	Weber number (-)
**Greek**	
*β*	contact angle (degree)
$\dot{\gamma }$	shear rate (s^−1^)
*σ*	surface tension coefficient (N/m)
*ρ_l_*	liquid density (kg/m^3^)
*ρ_g_*	gas density (kg/m^3^)
*η_c_*	characteristic viscosity (Pa·s)
*τ_y_*	yield stress (Pa)
